# Analysis of Hydroxy Fatty Acids from the Pollen of *Brassica campestris* L. var. *oleifera* DC. by UPLC-MS/MS

**DOI:** 10.1155/2013/874875

**Published:** 2012-10-10

**Authors:** Nian-Yun Yang, Yi-Fang Yang, Kun Li

**Affiliations:** Department of Traditional Chinese Medicine, Shanghai Institute of Pharmaceutical Industry, Shanghai 200040, China

## Abstract

Ultraperformance liquid chromatography coupled with negative electrospray tandem mass spectrometry (UPLC-ESI-MS/MS) was used to determine 7 hydroxy fatty acids in the pollen of *Brassica campestris* L. var. *oleifera* DC. All the investigated hydroxy fatty acids showed strong deprotonated molecular ions [M–H]^−^, which underwent two major fragment pathways of the allyl scission and the *β*-fission of the alcoholic hydroxyl group. By comparison of their molecular ions and abundant fragment ions with those of reference compounds, they were tentatively assigned as 15,16-dihydroxy-9Z,12Z-octadecadienoic acid (**1**), 10,11,12-trihydroxy-(7Z,14Z)-heptadecadienoic acid (**2**), 7,15,16-trihydroxy-9Z,12Z-octadecadienoic acid (**3**), 15,16-dihydroxy-9Z,12Z-octadecadienoic acid (**4**), 15-hydroxy-6Z,9Z,12Z-octadecatrienoic acid (**5**), 15-hydroxy-9Z,12Z- octadecadienoic acid (**6**), and 15-hydroxy-12Z-octadecaenoic acid (**7**), respectively. Compounds **3**, **5**, and **7** are reported for the first time.

## 1. Introduction

**Figure 1:**
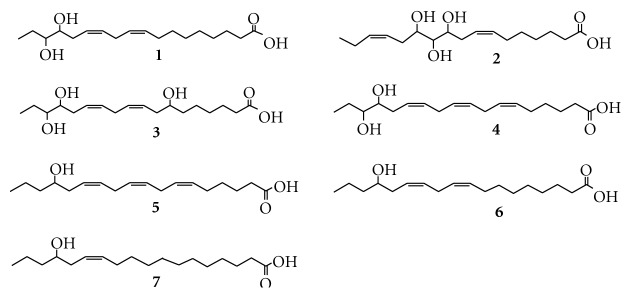
Structural formulas of hydroxy fatty acids in the present study.

Pollen contains many kinds of nutrient which are necessary to human body. It is well known as a natural nutrition, health food, and “perfect food.” The pollen preparation (Qian Lie Kang Tablets) made of *Brassica campestris *L. var.* oleifera *DC. has also been widely used in China as a good treatment for benign prostatic hyperplasia (BPH), and a chemical study has found plenty of fatty acids in the pollen [1]. Reports have shown promising therapeutic effects of fatty acids and their derivatives in treatment of BPH [2–6]. Hydroxy fatty acids contained in pollen are important bioactive substances. Various kinds of hydroxy fatty acids exhibit many pharmacological activities such as antitumor, antifungal, and prostaglandin E-like [7–9]. Although the pollen preparation has been used as phytotherapy for BPH for a long time, its active components and mechanism of action remain unclear. The effects of the supercritical fluid extract and its residue of the plant were screened so as to clarify its active constituents, and its supercritical fluid extract decreased the size of the prostate of androgen-induced prostatic rats by *in vivo* experiments (*P* < 0.01) and also demonstrated remarkable inhibitory effects on 5*α*-reductase and aromatase through *in vitro* experiments. The chemical investigation of its supercritical fluid extract has led to the isolation of some fatty acids and fatty acid derivatives [10], and the activity test experiments showed that fatty acids possessed strong inhibitory activity on 5*α*-reductase, which were consistent with literature reports [2–4]. The activity test experiments also displayed that fatty acid derivatives possessed strong inhibitory activity on aromatase [10]. 5*α*-reductase and aromatase are two important effect targets on prostate hyperplasia [11], so fatty acids and fatty acid derivatives play a coreaction role in treatment of BPH. UPLC-MS combine the efficient separation capability of UPLC and the great power in structural characterization of MS and provide new powerful approach to identify the constituents in plant extracts rapidly and accurately. In this paper, we investigated the fragmentation behaviors of hydroxy fatty acids in a Micromass Q/TOF Mass Spectrometer and emphasized on the structural determination of hydroxy fatty acids **1**–**7** (Figure 1) from the supercritical fluid extract by UPLC-ESI-MS/MS.

## 2. Materials and Methods

### 2.1. Standards and Reagents

Reference compounds, 15,16-dihydroxy-9Z,12*Z*-octadecadienoic acid (**1**) and 10,11,12-trihydroxy-(7*Z*,14*Z*)-heptadecadienoic acid (**2**), were isolated from the Pollen of *B*.* campestris *L. var.* oleifera* DC. by the authors. Their structures were unambiguously identified by NMR techniques [10], and their purities were above 95% as determined by HPLC. HPLC-grade acetonitrile (MeCN) and methanol (AR grade) were obtained from Labscan (Stillorgan, Ireland), and the water used for UPLC was purified by a Milli-Q system (Millipore, Milford, MA, USA). Ethanol for plant extraction was purchased from Shanghai Chemical Corporation (Shanghai, China).

### 2.2. Plant Material and Sample Preparation

The pollen of *B. campestris *L. var.* oleifera *DC. was collected from Inner Mongolia Autonomous Region of China in March 2004 and was identified by Professor Xu Feng of Jiangsu Botanic Institute. A voucher specimen (BC-20060726) was kept in the Herbarium of Shanghai Institute of Pharmaceutical Industry. The dried powder (1 kg) of the rape pollen, of which the cell wall was broken by zymolysis, was extracted in supercritical fluid CO_2_ with 95% ethanol as the cosolvent; a dark brown residue (52.3 g) was obtained. Two milligrams of the supercritical fluid extract were dissolved in 10 ml of methanol and filtered over 0.45 *μ*m microporous membrane for UPLC-MS analysis.

### 2.3. UPLC-MS Analysis

UPLC mass spectrometry was carried out on a Waters nanoACQUITY UPLC system combined with A Micromass Q/TOF Mass Spectrometer via an electrospray ionization interface. UPLC conditions are as follows: the column was an nanoACQUITY BEH130 C18 Column and the column temperature was maintained at 30°C; the mobile phase was a gradient elution which was mixed with solvents A (water) and B (acetonitrile). The gradient program was as follows: initial 0–2 min, using gradient elution A–B (98:2, v/v), 2–6 min, linear change to A–B (65:35, v/v), 6–12 min, linear change to A–B (50:50, v/v), 12–15 min, linear change to A–B (40:60, v/v), 15–18 min, linear change to A–B (20:80, v/v), and18–20 min, linear change to A–B (0:100, v/v); the sample injection volume was 1 *μ*L; the flow rate was 0.2 mL·min^−1^. The Q/TOF MS condition was set as follows: ionizing source was electrospray ionization (negative ion mode); drying gas (N_2_) flow rate was 10.0 L/min; drying gas temperature was 320°C; capillary voltage was set to 3000 V; fragmentation voltage was set to 120 V; the full-scan second order mass spectra of the investigated compounds from *m/z *50–350 Da were measured using 500 ms for collection time and three microscans were summed.

## 3. Results and Discussion

### 3.1. Mass Spectrometry Analysis of Reference Compounds 1-2

At first, the two reference compounds 15,16-dihydroxy-9Z,12*Z*-octadecadienoic acid (**1**), and 10,11,12-trihydroxy-(7*Z*, 14*Z*)-heptadecadienoic acid (**2**), were analyzed by UPLC-ESI-MS. Both hydroxy fatty acids showed strong [M–H]^−^ ions, which is similar to the hydroxy fatty acids reported in *Tydemania expeditionis* [12]. 

The full MS/MS product ion spectrum of compound **1** was shown in Figure 3. Six primary product ions are observed and it is proposed that they are formed in two major fragment pathways (Figure 4). Pathway I involves the *β*-fission of alcoholic hydroxyl group along with the neutral loss of propionaldehyde and 2-hydroxy-butyraldehyde or 1-hydroxy-2-butanone, which results in the formation of *m/z* 253 and *m/z* 223 as the major peaks. Pathway II involves the allyl scission leading to the formation of *m/z* 183 as the main peak. Some other pathways indicated neutral loss of H_2_O or CO or H_2_ from the deprotonated molecular and fragments. The allyl scission of the molecular ion [M–H]^−^ at *m/z *313 of **2** leads to the formation of *m/z* 213, and the *β* -fission of OH group of **2 **lost 1,3-pentadiene and 2,3-dihydoxy-octa-5-enal or 1,3-dihydoxy-octa-5-en-2-one to produce the major peaks of *m/z* 245 and *m/z* 155.

### 3.2. Structural Analysis of Hydroxy Fatty Acids in the Fingerprint Chromatogram

**Figure 2:**
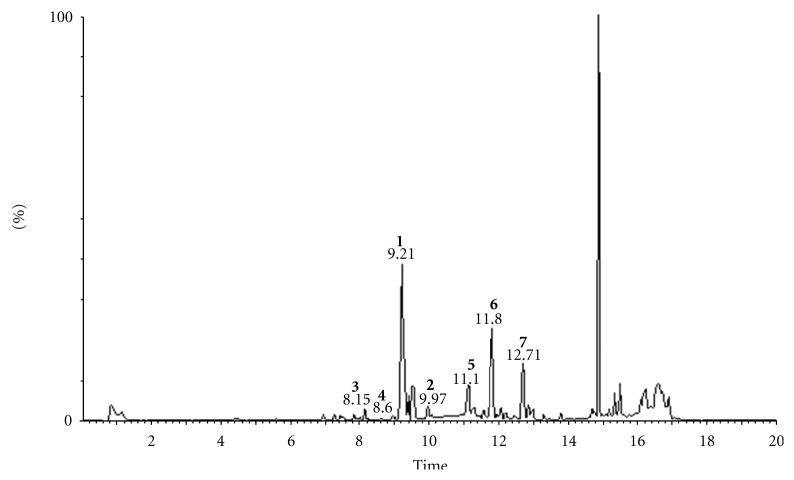
Total ion chromatogram of supercritical fluid extract of pollen of *Brassica campestris *L. var.* oleifera *DC.

**Figure 3:**
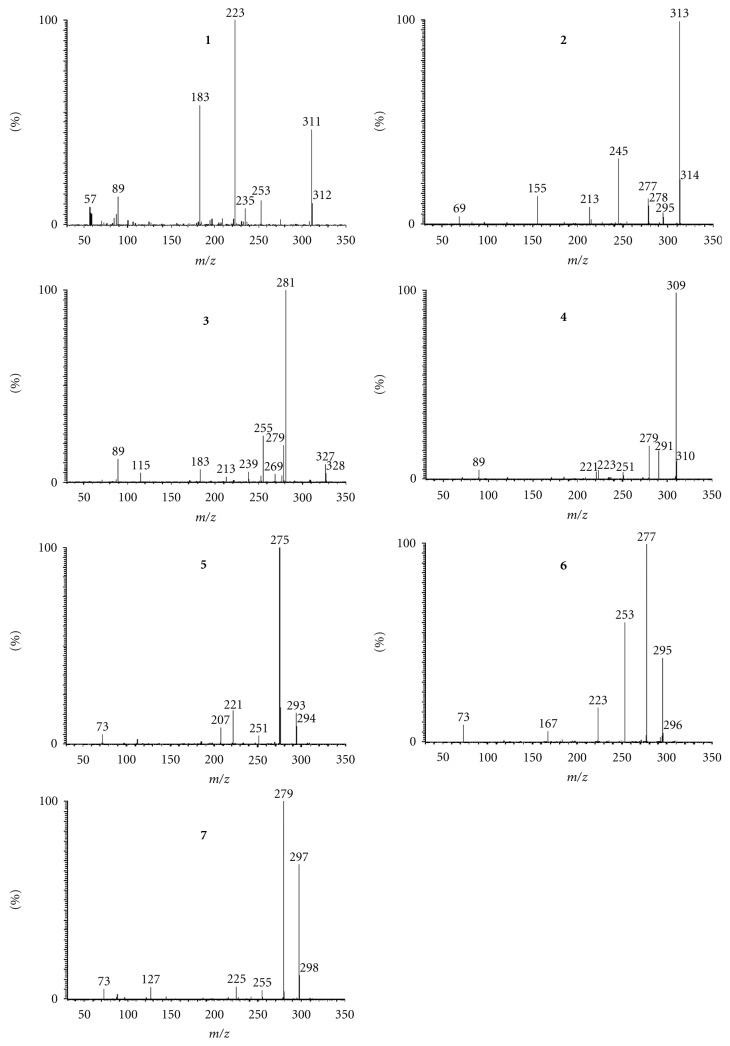
The product ion spectra of compounds **1**–**7**.

UPLC-ESI-MS was used to analyze the chemical constituents of the supercritical fluid extract of pollen of *B*.* campestris *L. var*. oleifera *DC. (Figure 2), and the fragmentation behaviour of 7 hydroxy fatty acids was analyzed by UPLC-ESI-MS/MS. In the total ion chromatograms (Figure 2), the peaks at 9.21 min and 9.97 min showed a molecular ion [M–H]^−^ at *m/z* 311 and 313, respectively (Figure 3), which were identical to compounds **1**-**2**, respectively and confirmed by the same characteristic data of UPLC analysis. In the total ion chromatograms, peak at 8.15 min showed a molecular ion [M–H]^−^ at *m/z* 327, the peak at 8.60 min showed a molecular ion [M–H]^−^ at *m/z* 309, the peak at 11.10 min showed a molecular ion [M–H]^−^ at *m/z* 293, the peak at 11.80 min showed a molecular ion [M–H]^−^ at *m/z* 295, and the peak at 12.71 min showed a molecular ion [M–H]^−^ at *m/z* 297, which were identified by UHPLC-MS/MS analysis. From the product ion spectra (Figure 3) of compound **3** at 8.15 min, compound **4** at 8.60 min, compound **5** at 11.10 min, compound **6** at 11.80 min, and compound **7** at 12.71 min, it was found that they showed almost the same fragment pattern with compounds **1**-**2**. 

**Figure 4:**
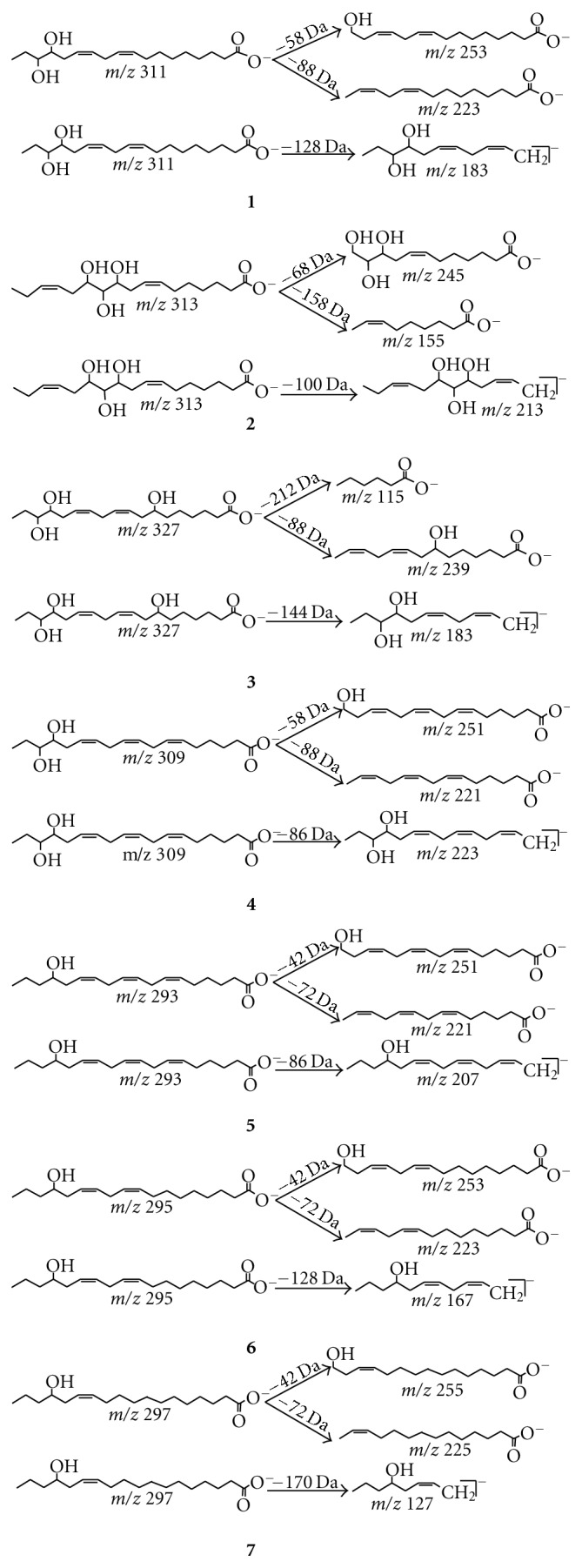
Characteristic fragmentation pathways for the molecular anion of compounds **1**–**7**.

The molecular ion of compound **3** indicated an excess of 16 Da in comparison with that of **1**, which suggested a surplus hydroxyl group in compound **2**. The full MS/MS product ion spectrum (Figure 3) of compound **3** showed the characteristic product ions of *m/z* 239, 183, 269, 115, and 89, which were formed from the allyl scission and the *β*-fission of OH group respectively (Figure 4). The fragment *m/z* 115 resulted from a *β*-fission of the OH group, which showed a hydroxyl group on C-7. Some other pathways also displayed loss of H_2_O or CO from the deprotonated molecular and fragments. The configuration of the olefinic bonds was deduced from the biosynthetic pathway, and the natural sources of unsaturated fatty acids and their derivatives are rich in the *cis* isomer, so the olefinic bonds of compound **3 **were Z geometry. Thus, compound **3** was tentatively assigned as 7,15,16-trihydroxy-9Z,12*Z*-octadecadienoic acid, and it is reported for the first time.

The full MS/MS product ion spectrum (Figure 3) of compound **4** showed the characteristic product ions of *m/z* 221, and 223, which were formed from the allyl scission, and another major peak of *m/z* 251 resulted from the *β*-fission of the OH group (Figure 4). These characteristic product ions suggested an additional olefinic bond at C-6 in compound **4 **in comparison with **1**. Some other pathways displayed loss of 18 Da or 30 Da from the deprotonated molecular and fragments. The configuration of the olefinic bonds was also determined from the biosynthetic pathway, and compound **4 **was tentatively assigned as 15,16-dihydroxy-9Z,12*Z*-octadecadienoic acid, and 15*S*,16*S*-**4** was reported in the literature [13].

The molecular ion of compound **5** indicated a lack of 16 Da in comparison with that of **4**. The full MS/MS product ion spectrum (Figure 3) of compound **5** showed the characteristic peaks of *m/z* 73, 207, 221 and 251. The fragment at *m/z* 251 resulted from the *β*-fission of OH group (Figure 4), which showed a hydroxyl group on C-15. The configuration of the olefinic bonds was also determined from the biosynthetic pathway, and compound **5 **was tentatively assigned as 15-hydroxy-6Z,9Z,12*Z*-octadecatrienoic acid, and it is reported for the first time.

The molecular ion of compound **6** indicated a lack of 16 Da in comparison with that of **1**. The full MS/MS product ion spectrum (Figure 3) of compound **6** showed the characteristic peaks of *m/z* 73, 167, 223, and 253. The fragment at *m/z* 253 resulted from the -fission of OH group (Figure 4), which showed a hydroxyl group on C-15. The configuration of the olefinic bonds was also determined from the biosynthetic pathway, and compound **6 **was tentatively assigned as 15-hydroxy-9Z,12*Z*-octadecadienoic acid, and 15*R*-**6** was reported in the literature [14].

The molecular ion of compound **7** indicated an excess of 2 Da in comparison with that of **6**. The full MS/MS product ion spectrum (Figure 3) of compound **7** showed the characteristic peaks of *m/z* 73, 127, 225, and 255. The fragment at *m/z* 255 resulted from the *β*-fission of OH group (Figure 4), which showed a hydroxyl group on C-15. The configuration of the olefinic bonds was also determined from the biosynthetic pathway, and compound **7** was tentatively assigned as 15-hydroxy-12*Z*-octadecaenoic acid, and it is reported for the first time.
